# Serum Levels of Advanced Glycation Endproducts and Other Markers of Protein Damage in Early Diabetic Nephropathy in Type 1 Diabetes

**DOI:** 10.1371/journal.pone.0035655

**Published:** 2012-04-25

**Authors:** Bruce A. Perkins, Naila Rabbani, Andrew Weston, Linda H. Ficociello, Antonysunil Adaikalakoteswari, Monika Niewczas, James Warram, Andrzej S. Krolewski, Paul Thornalley

**Affiliations:** 1 Division of Endocrinology, University of Toronto, Toronto, Canada; 2 Clinical Sciences Research Institute, Warwick Medical School, University of Warwick, Coventry, United Kingdom; 3 Section on Genetics and Epidemiology, Joslin Diabetes Center, Boston, Massachusetts, United States of America; Children's Hospital Boston/Harvard Medical School, United States of America

## Abstract

**Objective:**

To determine the role of markers of plasma protein damage by glycation, oxidation and nitration in microalbuminuria onset or subsequent decline of glomerular filtration rate (termed “early GFR decline”) in patients with type 1 diabetes.

**Methods:**

From the 1^st^ Joslin Kidney Study, we selected 30 patients with longstanding normoalbuminuria and 55 patients with new onset microalbuminuria. Patients with microalbuminuria had 8–12 years follow-up during which 33 had stable GFR and 22 early GFR decline. Mean baseline GFR_CYSTATIN C_ was similar between the three groups. Glycation, oxidation and nitration markers were measured in protein and ultrafiltrate at baseline by liquid chromatography-tandem mass spectrometry using the most reliable methods currently available.

**Results:**

Though none were significantly different between patients with microalbuminuria with stable or early GFR decline, levels of 6 protein damage adduct residues of plasma protein and 4 related free adducts of plasma ultrafiltrate were significantly different in patients with microalbuminuria compared to normoalbuminuria controls. Three protein damage adduct residues were decreased and 3 increased in microalbuminuria while 3 free adducts were decreased and one increased in microalbuminuria. The most profound differences were of N-formylkynurenine (NFK) protein adduct residue and N_ω_-carboxymethylarginine (CMA) free adduct in which levels were markedly lower in microalbuminuria (P<0.001 for both).

**Conclusions:**

Complex processes influence levels of plasma protein damage and related proteolysis product free adducts in type 1 diabetes and microalbuminuria. The effects observed point to the possibility that patients who have efficient mechanisms of disposal of damaged proteins might be at an increased risk of developing microalbuminuria but not early renal function decline. The findings support the concept that the mechanisms responsible for microalbuminuria may differ from the mechanisms involved in the initiation of early renal function decline.

## Introduction

Diabetic nephropathy is the single most important cause of renal failure in North America. Patients with type 1 diabetes face a one in three lifetime risk of developing macroalbuminuria. Despite the widespread adoption of renoprotective therapies, these patients remain at high risk for development of end stage renal disease [1]. Identification of risk at the earliest stages of diabetic nephropathy and identification of the pathological processes that lead to early loss of glomerular filtration rate (GFR) may permit the development and implementation of interventions at a stage when they are most likely to be effective.

The traditional concept of diabetic nephropathy held that microalbuminuria (as the fundamental early prognostic variable) heralds macroalbuminuria [2]–[4], which after long-term exposure initiates the process of decline in GFR that leads to end stage-renal disease [5]. However, two key refinements to this concept arose from systematic longitudinal study. First, contrary to the traditional model, microalbuminuria proved to be a dynamic process that was more likely to remit to normal albumin excretion (‘normoalbuminuria’) than to progress to macroalbuminuria [6], [7]. Second, independent of subsequent progression or remission of microalbuminuria, approximately one third of patients with new onset microalbuminuria begin a linear process of early GFR decline many years before the onset of macroalbuminuria [8], [9]. This new concept emphasized the prognostic importance of the two early markers of diabetic nephropathy: a) the new onset of microalbuminuria rather than its subsequent trajectory, and b) the initiation of early GFR decline. These phenotypes may occur concurrently and subsequently progress at different rates. Improved understanding of the mechanisms of development of both microalbuminuria and early GFR decline, identification of related prognostic biomarkers and therapeutic interventions will likely lead to improved treatment of diabetic nephropathy [10].

Proteins suffer increased damage by glycation, oxidation and nitration in diabetes [11]–[17]. Increased damage to glomerular proteins was found in experimental diabetes linked to the development of early-stage diabetic nephropathy [18]–[21]. Glycation, oxidation and nitration of plasma protein and related glycation, oxidation and nitration free adducts – glycated, oxidized and nitrated amino acids formed by proteolysis of glycated, oxidized and nitrated proteins, were increased in clinical diabetes [20], [22], [23]. Therapeutic agents that induce remission of microalbuminuria in clinical diabetes – such as angiotensin receptor blockers and high dose thiamine [24], [25]– decreased levels of damage to glomerular and plasma protein and or levels and flux of related free adducts [18], [26]. We hypothesised that plasma markers of protein damage, glycated, oxidized and nitrated plasma proteins or related free adducts – glycated, oxidized and nitrated amino acids formed by the proteolysis of plasma and other proteins - may be part of the causal pathway for the development of microalbuminuria and/or early GFR decline.

Proteins in human tissues, plasma and other body fluids suffer continual spontaneous damage by glycation, oxidation and nitration in vivo. Important glycation adducts in vivo are the early-stage glycation adduct, fructosyl-lysine (FL), and advanced glycation endproducts (AGEs). Important AGEs quantitatively are hydroimidazolones derived from arginine residues modified by glyoxal, methylglyoxal and 3-deoxyglucosone, G-H1, MG-H1 and 3DG-H (and related structural isomers), respectively [22]. Other important and widely-studied AGEs are N_ε_-carboxymethyl-lysine (CML), N_ω_-carboxymethyl-arginine (CMA), N_ε_-carboxyethyl-lysine (CEL) and pentosidine (PENT). Markers of oxidative damage to proteins are: methionine sulfoxide (MetSO), dityrosine (DT) and N-formylkynurenine (NFK) – products of oxidation of tyrosine and tryptophan residues, respectively. A widely-studied marker of nitration damage to proteins is 3-nitrotyrosine (3-NT) [27].

In previous reports of the 1^st^ Joslin Study of the Natural History of Microalbuminuria in Type 1 diabetes we detailed the risk of early GFR decline [8], [9], [28], [29]. Selecting from this cohort patients with normoalbuminuria and patients with new onset microalbuminuria with or without early GFR decline over 10 or more years of follow-up, we study the association of plasma protein glycation, oxidation and nitration with the development of microalbuminuria and of early GFR decline.

## Methods

The protocol and consent procedures were approved by the Committee on Human Studies of the Joslin Diabetes Center. Written informed consent was obtained from all participants.

### Study Subjects

Among the 943 patients with normoalbuminuria enrolled in the 1^st^ Joslin Study of the Natural History of Microalbuminuria in Type 1 Diabetes (“The 1^st^ Joslin Kidney Study”), microalbuminuria developed in 109 during the first four years of observation [6], [30]. Eighty-six of the 109 patients (79%) were followed for greater than 10 years after the onset of microalbuminuria and they were available for this study. We ranked them according to the slope of the glomerular filtration rate estimated by serum cystatin C (GFR_CYSTATIN C_) over time, expressed as percent change per year. The protocol used for assessment of early GFR decline has been described previously [8]. In brief, patients had on average 5.6±1.9 serum Cystatin C determinations. GFR_CYSTATIN C_ in ml/min was approximated using the concentration of serum cystatin C (in mg/L) and application of a formula developed by MacIsaac et al. for patients with diabetes [31]. The slope of GFR_CYSTATIN C_, expressed as percent change per year, was derived from the linear regression of log-transformed values for each individual. As previously published, a negative slope or trend in renal function change greater than 3.2 percent per year qualified as an abnormal rate of loss (designated ‘*early GFR decline*’) [8], [32]. We selected 22 subjects with early GFR decline and 33 subjects with stable GFR_CYSTATIN C_ in whom the subsequent change in renal function did not decline beyond a slope of −3.1 percent per year. The microalbuminuria cases with stable GFR had a subsequent median slope of −0.95 percent per year [interquartile range, −1.61 to −0.43] compared to −4.87 [interquartile range, −11.83 to −3.78] in the microalbuminuria cases with early GFR decline subgroup (p<0.0001).

#### Selection of Normoalbuminuria Controls

From the 650 patients participating in the 1^st^ Joslin Kidney Study who remained normoalbuminuric during follow-up we randomly selected 30 individuals who did not have onset of microalbuminuria over the course of 12 years of follow-up as controls for comparison with the microalbuminuria cases [33]. These subjects were followed for a mean of 12.5±1.9 years from baseline and serum cystatin C for estimation of GFR_CYSTATIN C_ was obtained an average of 3.6±1.5 times. The median slope of GFR_CYSTATIN C_ was −0.88 percent per year [interquartile range, −1.18 to −0.67] (comparison with microalbuminuria cases, p<0.0001). None of the normoalbuminuria controls had early GFR decline.

#### Measurement of protein glycation, oxidation and nitration adducts

Plasma specimens obtained within the first 4 years of onset of microalbuminuria and within the first 4 years of follow-up in patients with normoalbuminuria were selected for the study. Protein glycation, oxidation and nitration adducts were determined by LC-MS/MS with quantitation by stable isotopic dilution analysis from stored plasma samples frozen at −85°C [23]. Eleven analytes were determined: FL, G-H1, MG-H1, 3DG-H, CML, CMA, CEL, MetSO, NFK, DT and 3-NT by stable isotopic dilution analysis LC-MS/MS, and PENT by concurrent fluorescence detection. They were quantified by reference to calibration curve response of authentic standards. LC-MS/MS and fluorescence detection was performed with a Waters Acquity UPLC system with Acquity fluorescence detector and Quattro Premier XE tandem mass spectrometer. Plasma glycation, oxidation and nitration free adducts were determined by direct analysis of plasma ultrafiltrates (12 kDa cut-off). Glycation, oxidation and nitration adduct residues of plasma protein were determined after exhaustive enzymatic hydrolysis by consecutive incubation with pepsin, pronase E, and finally aminopeptidase and prolidase, under nitrogen [23]. Sample identity was blinded from the investigator.

### Statistical Methods

All statistical analyses were performed in SAS version 9.1 (SAS Institute, Carey, North Carolina). For the baseline clinical variables other than urinary albumin excretion, comparisons between normoalbuminuria (n = 30) and microalbuminuria (n = 55) study groups as well as the comparison between microalbuminuria with stable GFR (n = 33) and the microalbuminuria with early GFR decline (n = 22) used means and Student's t-tests for continuous variables and percentages and χ^2^ tests for categorical variables. For urinary albumin excretion and the plasma free adduct concentrations, two-way comparisons were made in the log-scale using Wilcoxon rank sum tests and the trend across the three groups were made by Kruskal-Wallis tests. Longitudinal measures of log-transformed GFR_CYSTATIN C_ levels were analyzed using a general linear model which allowed for the derivation of subject-specific slopes (and subsequently percent changes per year). To account for the multiple comparisons owing to inclusion of 12 independent hypotheses on the single dataset (12 adducts were included in each of the analyses of adduct residues and free adducts), we maintained the family-wise error rate by considering statistical significance for these tests at α-level<0.0042 using the simple Bonferroni correction method of 0.05/12. Correlation among the adduct residues and among the free adducts associated with microalbuminuria were analyzed to determine redundancy of analytes and association with the slope of GFR_CYSTATIN C_ using Spearman correlation coefficients.

## Results

To investigate factors associated with the pathophysiology of the initiation of renal function loss, the study groups were derived from a population composed of patients whose onset of microalbuminuria was documented in the 1st Joslin Study of the Natural History of Microalbuminuria in Type 1 Diabetes. Selected participants has follow-up examinations spanning at least 10 to 12 yr after microalbuminuria onset for determination of the rate of GFR decline (slopes of GFR_CYSTATIN C_) and also had stored plasma available for analysis of the markers of protein damage. Subjects with new onset microalbuminuria were subdivided into 22 case patients with early GFR decline, defined as a decline of GFR_CYSTATIN C_ slope exceeding 3.2 percent per year, and a subgroup of 33 control subjects with stable GFR. The baseline clinical characteristics of these subgroups, compared with the longstanding normoalbuminuria group are shown in Table 1 according to microalbuminuria and early GFR decline status. Statistical comparisons were made in two ways: first, between patients with normoalbuminuria and microalbuminuria; and secondly between microalbuminuria patients with subsequent stable GFR or with early decline in GFR. Patients with microalbuminuria had a lower age, a higher proportion of smokers, a higher A1c during the baseline interval, and, by definition, higher urinary albumin excretion than patients with normoalbuminuria. They also had GFR_CYSTATIN C_ similar to the normoalbuminuria controls. Patients with microalbuminuria with early decline in GFR had higher A1c, total cholesterol and triglycerides compared to patients with microalbuminuria and stable GFR. These two groups did not differ in age, proportion of smokers, or levels of urinary albumin excretion at baseline. Furthermore, the levels of serum cystatin C or GFR_CYSTATIN C_ were similar at baseline in microalbuminuria cases regardless of subsequent stable GFR or early GFR decline.

**Table 1:** Baseline Clinical Characteristics of the 85 Type 1 Diabetes Subjects classified by presence or absence of Microalbuminuria and Early Decline in GFR.

Baseline Characteristic	Normo-albuminuria Group (N = 30)	Microalbuminuria Group (N = 55)		P for Group Comparison NA vs. MA	P for Subgroup Comparison* Stable vs. Early GFR Decline	P for trend
		Stable GFR Subgroup (n = 33)	Early GFR Decline Subgroup (n = 22)			
Male sex (%)	12 (40%)	17 (52%)	9 (41%)	0.52	0.44	0.60 (χ^2^)
Age (years)	41.9±6.2	36.3±8.8	37.8±8.0	0.005	0.51	0.03
Duration of Diabetes (years)	26.3±8.3	23.0±8.2	23.6±8.3	0.10	0.80	0.30
Current Smoking (%)	1 (3%)	13 (39%)	11 (50%)	<0.001	0.44	0.0004(χ^2^)
Systolic Blood Pressure (mmHg)	118.4±11.0	121.6±14.9	128.3±20.1	0.18	0.19	0.08
Diastolic Blood Pressure (mmHg)	73.4±6.2	76.0±10.5	76.6±8.7	0.25	0.82	0.36
Glycated Hemoglobin A_1C_ (percent)†	7.81±1.25	8.73±1.10	9.92±1.48	<0.001	<.001	<0.0001
Total Cholesterol (mg/dL)†	190±34	187±40	223±39	0.22	0.002	0.004
Triglycerides (mg/dL)†	107±97	97±56	157±117	0.71	0.02	0.05
Urinary Albumin Excretion Rate (µg/min)†						
2-year Baseline Interval^‡^	11.9 [9.7, 12.3]	45.1 [32.4, 58.2]	47.1 [32.8, 55.1]	<0.001	0.72	<0.0001
At Time of Adduct Measurement	6.4 [3.4, 13.6]	74.3 [35.2, 66.8]	59.8 [36.5, 85.5]	<0.001	0.19	<0.001
Serum Cystatin C (mg/L)	0.72±0.08	0.77±0.13	0.85±0.27	0.04	0.11	0.15
GRF_CYSTATIN C_ (m/min/1.73 m^2^)	117.1±13.6	111.9±18.3	104.6±25.8	0.07	0.22	0.15

Values are means ± standard deviation or median [interquartile range].

P-values for two-way comparisons were obtianed by Wilcoxon rank sum tests, and p-values for the trend across the three groups were made by Kruskal-Wallis tests.

* Comparison made among the two microalbuminuria subgroups.

† These values represent the mean values for all measurements taken during the two-year baseline interval used for classification of new onset microalbuminuria. To convert values for total cholesterol to millimoles per liter, multiply by 0.02586. To convert values for triglycerides to millimoles per liter, multiply by 0.01129.

Median levels of A1c in the three study groups of samples in which protein damage markers were measured are given in Table 2. A1c levels were similar to those in the mean baseline interval levels shown in Table 1. A1c was higher in patients with microalbuminuria compared to normoalbuminuria controls (P<0.001) and A1c was higher in patients with microalbuminuria and early decline in GFR compared to patients with microalbuminuria and stable GFR (P = 0.002).

**Table 2:** Median (and interquartile range) of Plasma Protein Content of Glycation, Oxidation and Nitration **Adduct Residues**.

	NA (n = 30)	MA (n = 55)		P for Group ComparisonNA vs. MA*	P for Subgroup Comparison Stable vs. Early GFR Decline*	P for Trend*
ADDUCT RESIDUES		Stable GFR (n = 33)	Early GFR Decline (n = 22)			
**Glycated Hemoglobin A1c** †	7.7[6.9, 8.3]	8.5 [7.7, 9.7]	10.1 [8.8, 11.2]	<0.001	0.002	<0.0001
***Monolysyl AGEs*** ‡						
FL (mmol/mol lys)	6.05 [5.24, 7.70]	4.78 [3.38, 6.11]	5.03 [4.31, 6.69]	0.004	0.19	0.01
CML (mmol/mol lys)	0.107 [0.0951, 0.142]	0.0884 [0.0723, 0.106]	0.0801[0.0664, 0.0957]	0.016	0.30	0.001
CEL (mmol/mol lys)	0.0117 [0.0084, 0.0189]	0.0445 [0.0121, 0.126]	0.042[0.0253, 0.195]	<0.001	0.55	0.001
***Fluorescent AGEs*** ‡						
PENT (mmol/mol lys)	0.000572 [0.000447, 0.000667]	0.000818 [0.00057, 0.00257]	0.000691[0.000523, 0.000916]	0.001	0.29	0.01
***Hydro-Imidazolone AGEs*** ‡						
G-H1 (mmol/mol arg)	0.0797 [0.0566, 0.111]	0.315[0.197, 0.477]	0.351[0.238, 0.393]	<0.001	0.81	<0.0001
MG-H1 (mmol/mol arg)	0.32 [0.27, 0.48]	0.49 [0.39, 0.61]	0.45 [0.39, 0.48]	0.057	0.04	0.002
3DG-H (mmol/mol arg)	0.24 [0.19, 0.28]	0.31 [0.22, 0.38]	0.33 [0.10, 0.37]	0.37	0.27	0.37
CMA (mmol/mol arg)	0.38 [0.33, 0.53]	0.33 [0.26, 0.45]	0.32 [0.26, 0.40]	0.011	0.49	0.03
***Oxidation Adducts***						
MetSO (mmol/mol met)	43.5 [39.5, 50.6]	45.1 [30.7, 63.4]	31.8 [27.4, 108]	0.60	0.62	0.35
NFK (mmol/mol trp)	0.688 [0.572, 0.809]	0.0921 [0.0604, 0.123]	0.0819 [0.0675, 0.113]	<0.001	0.40	<0.0001
DT (mmol/mol tyr)	0.0131 [0.00807, 0.0184]	0.0104 [0.00541, 0.0216]	0.00783 [0.00596, 0.0178]	0.44	0.83	0.29
***Nitration Adduct***						
3-NT (mmol/mol tyr)	0.00879 [0.00592, 0.0128]	0.00431[0.00322, 0.00718]	0.00337 [0.00254, 0.00423]	<0.001	0.14	<0.0001

* P-values for two-way comparisons were obtianed by Wilcoxon rank sum tests, and p-values for the trend across the three groups were made by Kruskal-Wallis tests.

† Values represent the measurement taken in the sample used for measurement of plasma free adducts. These values differ from those presented in Table 1 as the latter represent the mean values over the two-year baseline interval used for classification of new onset microalbuminuria.

‡ AGEs: Advanced Glycation endproducts.

NA, Normoalbuminuria. MA, Microalbuminuria. GFR, glomerular filtration rate.

FL, N_ε_-Fructosyl-lysine. CML, N_ε_-Carboxymethyl-lysine. CEL, N_ε_-(1-Carboxyethyl)lysine. PENT, Pentosidine. G-H1, N_δ_-(5-hydro-4-imidazolon-2-yl) ornithine. MG-H1, N_δ_-(5-hydro-5-methyl-4-imidazolon-2-yl)ornithine. 3DG-H, N_δ_-(5-hydro-5-(2,3,4-trihydroxybutyl)-4-imidazolon-2-yl)ornithine and related structural isomers, .

CMA, N_ω_-carboxymethylarginine. MetSO, methionine sulfoxide, NFK, N-formylkynurenine. DT, o,o′-dityrosine. 3NT, 3-nitrotyrosine.

### Glycation, oxidation and nitration adduct residues of plasma protein and related plasma free adducts

**Figure 1:**
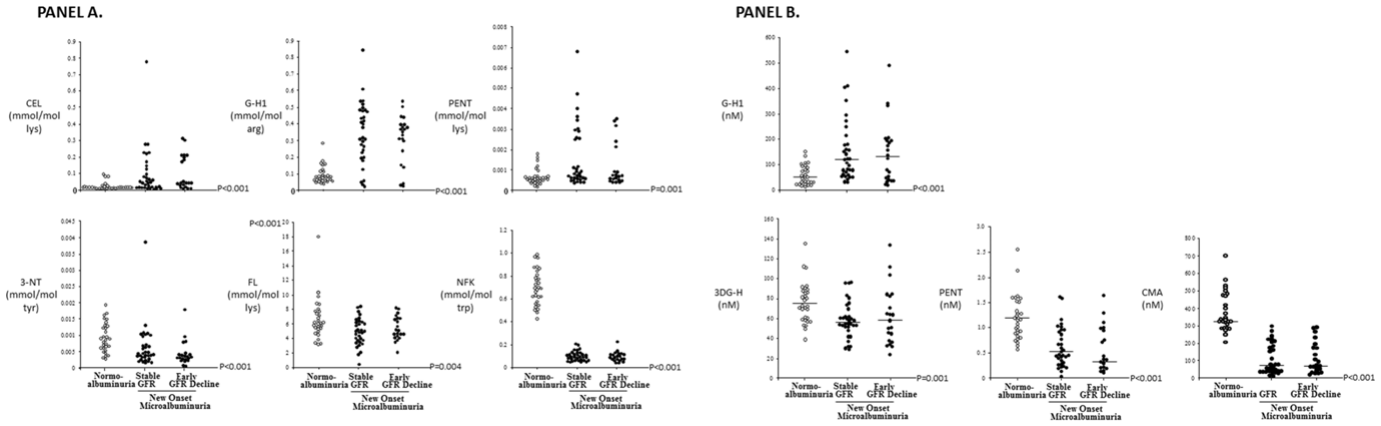
Comparison of Adduct Residue (Panel A) and Free Adduct (Panel B) Concentration in Cases of New Onset Microalbuminuria compared to Normoalbuminuria Controls. P values indicate the differences between Microalbuminuria Cases versus Normoalbuminuria Controls for the log-transformed adduct residue concentrations in the Wilcoxon Rank-Sum tests.

Plasma protein contents of FL, NFK and 3-NT adduct residues were lower in patients with microalbuminuria compared to normoalbuminuria controls. In contrast to this, plasma protein contents of G-H1, CEL and PENT residues were higher in patients with microalbuminuria compared to controls. There was no significant difference in the plasma protein content of glycation, oxidation and nitration adduct residues between patients with microalbuminuria with and without early GFR decline, as shown in Table 2 and Figure 1, Panel A.

For related free adducts, the plasma concentrations of 3DG-H, CMA and PENT free adducts were lower in patients with microalbuminuria compared to normoalbuminuria controls, whereas the plasma concentration of G-H1 free adduct was higher in patients with microalbuminuria compared to controls. There was no significant difference in the plasma concentrations of glycation, oxidation and nitration adducts residues between patients with microalbuminuria with and without early GFR decline, as shown in Table 3 and Figure 1, Panel B.

**Table 3:** Median (and interquartile range) of Plasma Concentration of Protein Glycation, Oxidation and Nitration **Free Adducts**.

	NA (n = 30)	MA (n = 55)		P for Group ComparisonNA vs. MA*	P for Subgroup Comparison Stable vs. Early GFR Decline*	P for Trend*
FREE ADDUCT		Stable GFR (n = 33)	Early GFR Decline (n = 22)			
***Monolysyl AGEs*** †						
FL (nM)	282 [180, 416]	262 [170, 332]	252 [183, 439]	0.74	0.14	0.39
CML (nM)	97 [76, 123]	74 [63, 89]	84 [52, 115]	0.02	0.54	0.05
CEL (nM)	40 [ 34, 56]	36 [ 29, 49]	42 [32, 52]	0.07	0.23	0.14
***Fluorescent AGEs*** †						
PENT (nM)	1.2 [0.9, 1.5]	0.5 [0.3, 0.9]	0.3 [0.2, 0.9]	<.0001	0.67	<0.0001
***Hydro-Imidazolone AGEs*** †						
G-H1 (nM)	51 [30, 88]	120 [63, 180]	131 [41, 194]	<.0001	0.49	0.0002
MG-H1 (nM)	484 [255, 710]	339 [263, 567]	297 [202, 510]	0.11	0.90	0.29
3DG-H (nM)	75 [59, 89]	55 [52, 62]	58 [38, 84]	0.001	0.66	0.004
CMA (nM)	330 [288, 471]	70 [37, 182]	65 [40, 156]	<.0001	0.86	<0.0001
***Oxidation Adducts***						
MetSO (nM)	988 [826, 1639]	806 [594, 1294]	850 [502, 1269]	0.01	0.31	0.04
NFK (nM)	27 [21,34]	22 [18,29]	22 [18,25]	0.39	0.60	0.09
DT (nM)	1.7 [1.3, 2.0]	1.4 [0.9, 2.1]	1.8 [1.5, 2.0]	0.85	0.09	0.39
***Nitration Adduct***						
3-NT (nM)	0.7 [0.6, 0.8]	0.8 [0.6, 1.3]	0.9 [0.5, 1.4]	0.02	0.72	0.14

* P-values for two-way comparisons were obtianed by Wilcoxon rank sum tests, and p-values for the trend across the three groups were made by Kruskal-Wallis tests.

† AGEs: Advanced glycation endproducts.

NA, Normoalbuminuria. MA, Microalbuminuria. GFR, glomerular filtration rate.

FL, N_ε_-Fructosyl-lysine. CML, N_ε_-Carboxymethyl-lysine. CEL, N_ε_-(1-Carboxyethyl)lysine. PENT, pentosidine, G-H1, N_δ_-(5-hydro-4-imidazolon-2-yl) ornithine, MG-H1, N_δ_-(5-hydro-5-methyl-4-imidazolon-2-yl)-ornithine, 3DG-H, N_δ_-(5-hydro-5-(2,3,4-trihydroxybutyl)-4-imidazolon-2-yl)ornithine and related structural isomers.

CMA, N_ω_-carboxymethylarginine. MetSO, methionine sulfoxide. NFK, N-formylkynurenine. DT, o,o′-dityrosine. 3NT, 3-nitrotyrosine.

Among the 6 adduct residues with differences between microalbuminuria cases and normoalbuminuria controls, we sought to determine if there was systematic redundancy of their associations with A1c, with each other, and with the slope of GFR_CYSTATIN C_ by way of a correlation matrix. Those adduct residues that shared a Spearman correlation coefficient whose magnitude exceeded 0.5 were highlighted in bold font.We found that none of the adduct residues shared substantial correlation with A1c. However, substantial correlation was observed between only 4 of the 21 comparisons in the matrix, suggesting minimum redundancy of each individual marker: 3-NT shared association with NFK, CEL, and GH1 (spearman correlation coefficients 0.51, −0.52, and −0.54, respectively) while CEL and GH1 shared a correlation coefficient of 0.70 (Table 4). Similarly, in the correlation matrix demonstrating the associations between the 4 free adducts with differences between microalbuminuria cases and normoalbuminuria controls we observed association only between CMA and GH1 (Table 5). Consistent with the lack of association of markers of protein damage with cases of early GFR decline as compared to controls with stable GFR, we also did not observe substantial association in the rate of GFR loss – represented by the slope of GFR_CYSTATIN C_ – with protein adducts or adduct residues, shown in the last rows of Table 4 and Table 5, respectively.

**Table 4:** Matrix of Correlation Between the Adduct Residues with Significant Differences Between Microalbuminuria Cases and Normoalbuminuria Controls.

	GLYCA1C	NFK	3NT	FL	CEL	GH1	PENT	Slope of GFR_CYSTATIN C_ *
**GLYCA1C**	1							
**NFK**	−0.45, p<0.0001	1						
**3NT**	−0.25, p = 0.02	**0.51, p<0.0001**	1					
**FL**	0.14, p = 0.20	0.11, p 0.32	0.27, p 0.014	1				
**CEL**	0.17, p = 0.12	−0.35, p = 0.001	**−0.52, p<0.0001**	0.05, p = 0.68	1			
**GH1**	0.29, p = 0.008	−0.44, p<0.0001	**−0.54, p<0.0001**	0.01, p = 0.92	**0.70, p<0.0001**	1		
**PENT**	0.09, p = 0.42	0.04, p = 0.71	0.28, p = 0.01	−0.24, p = 0.03	−0.07, p = 0.51	−0.03, p = 0.77	1	
**Slope of GFR_CYSTATIN C_** *	−0.38, p = 0.0004	0.28, p = 0.01	0.3, p = 0.005	0.06, p = 0.58	−0.25, p = 0.02	−0.27, p = 0.01	−0.06, p 0.59	1

The top number in each cell is the Spearman Correlation Coefficient ρ, while the bottom number is the p-value obtained from testing the hypothesis ρ = 0. P-values are not applicable for entries on the diagonal. Spearman Correlation Coefficients with magnitude ≥0.5 are indicated in bold.

* Slope is reported by percent change per year, as described in Methods.

**Table 5:** Matrix of Correlation Between the Free Adducts with Significant Differences Between Microalbuminuria Cases and Normoalbuminuria Controls.

	GLYCA1C	CMA	PENT	3DGH	GHI	Slope of GFR_CYSTATIN C_ *
**GLYCA1C**	1					
**CMA**	−0.47, p<0.0001	1				
**PENT**	−0.2, p = 0.07	0.33, p 0.002	1			
**3DGH**	−0.26, p = 0.016	0.25, p = 0.02	0.33, p 0.002	1		
**GH1**	0.11, p = 0.34	**−0.52, p<0.0001**	−0.11, p = 0.33	0.24, p = 0.03	1	
**Slope of GFR_CYSTATIN C_** *	−0.38, p = 0.0004	0.29, p = 0.008	0.21, p = 0.05	0.06, p = 0.61	−0.15, p 0.18	1

The top number in each cell is the Spearman Correlation Coefficient ρ, while the bottom number is the p-value obtained from testing the hypothesis ρ = 0. P-values are not applicable for entries on the diagonal. Spearman Correlation Coefficients with magnitude ≥0.5 are indicated in bold.

* Slope is reported by percent change per year, as described in Methods.

## Discussion

In the analysis of a type 1 diabetes cohort with normoalbuminuria followed longitudinally for the development of microalbuminuria and subsequent early GFR decline, we found a striking association of plasma protein contents of 6 adduct residues and plasma concentrations of 4 free adducts of protein damage with the new onset of microalbuminuria. Contrary to the prevailing assumptions about plasma markers of protein damage, the majority had lower levels at the new onset of microalbuminuria as compared to those with longstanding normoalbuminuria irrespective of subsequent GFR changes. These markers were generally either not associated or inversely correlated with levels of A1c, and they did not strongly associate with each another.

The plasma protein content of glycation, oxidation and nitration adduct residues relates positively to the in situ rate of glycation, oxidation and nitration of plasma protein and negatively to the rate of plasma clearance and repair of damaged plasma proteins. In previous work, it was observed that the levels of glycation, oxidation and nitration adduct residues of plasma protein of patients with type 1 diabetes and normoalbuminuria were higher compared to healthy controls: FL, CEL, G-H1, MG-H1, 3DG-H, PENT, MetSO, NFK and 3-NT were all 2–4-fold higher [20]. In the current analysis, however, we evaluated changes in plasma protein damage markers in patients with type 1diabetes with and without new onset microalbuminuria, and in microalbuminuria the effect of subsequent early GFR decline. In previous studies of patients with type 1 diabetes and normoalbuminuria, higher plasma protein content of FL correlated strongly with higher A1c [20]. In our cohort we found that patients with new onset microalbuminuria had greater glycemic exposure, as represented by higher levels of A1c, compared to patients with longstanding normoalbuminuria. It might be expected that FL and AGE content of plasma protein are higher in patients with new onset microalbuminuria – related to increased exposure to plasma glucose concentration and downstream formation of FL and AGEs [20]. To the contrary, we see a strong pattern of lower levels of circulating adduct residues for FL, 3-NT and NFK. Decreased content of FL residues likely reflects increased selective clearance of FL-modified albumin as the glomerular filter widens. Supporting this, an increased FL residue content of albumin excreted in urine of patients with diabetes and microalbuminuria compared to patients with diabetes and normoalbuminuria has been previously observed [21]. Such an observation implies that the mechanism for our observed higher circulating levels of adduct residues may be an increased clearance of those markers. If indeed microalbuminuria is associated with increased clearance of FL-modified albumin, the use of agents that decrease intra-glomerular pressure such as angiotensin receptor blockade would be anticipated to attenuate such clearance. Such increased retention of FL-modified albumin has been observed in a study that administerd angiotensin receptor blockade to patients with type 2 diabetes and microalbuminuria [26]. As the use of renin-angitoensin-aldosterone system inhibition such as angiotensin receptor blockade was not observed in this new onset microalbuminuria cohort assembled in the early 1990's, the finding of lower levels of the three adduct residues in the microalbuminuric subjects may be explained by an increase in their clearances. The lower levels of NFK and 3-NT adduct residues in plasma protein may additionally be due to decreased oxidative degradation of FL residues in plasma protein. The observation of higher plasma protein G-H1, CEL, and pentosidine residue content in patients with microalbuminuria is likely explained by increased exposure of plasma protein to the precursor glycating agents glyoxal, methylglyoxal and pentose-derived dicarbonyls, respectively. This may arise from decreased quality of glycemic control - as indicated by positive correlations of plasma protein G-H1, CEL, and pentosidine residue content with A1c.

Free adducts of protein glycation, oxidation and nitration in plasma originate from proteolysis of glycated, oxidized and nitrated proteins in tissues, plasma and other body fluids. The levels of plasma glycation, oxidaton and nitration free adducts relate positively to whole body flux of glycation, oxidation and nitration of proteins and rates of tissue proteolysis, and negatively to glycation, oxidation and nitration adduct repair and renal clearance. The levels of plasma glycation and oxidation free adducts of patients with type 1 diabetes and normoalbuminuria were increased compared to healthy controls. Specifically, free adducts of FL, CML, CEL, G-H1, MG-H1, 3DG-H and MetSO were all increased from 2–10-fold [20]. As with the plasma protein adduct residue described above, we also assessed differences in the free adduct plasma protein damage markers in patients with type 1diabetes with and without new onset microalbuminuria. The increased G-H1 free adduct in new onset microalbuminuria may relate to increased flux of formation of G-H1-modified protein, feeding through to increased plasma G-H1 concentration by proteolysis. Decreases in plasma concentrations of 3DG-H, pentosidine and CMA in new onset microalbuminuria, compared to normoalbuminuria controls, are unlikely to reflect decreased flux of formation of these adducts as glucose exposure was higher. Neither are these changes likely to reflect increased adduct repair - as no repair processes are known - or to reflect inhibition of tissue proteolysis as in this case all protein damage free adducts would be expected to be lower. Rather these decreases may reflect increased renal clearance of 3DG-H, PENT and CMA free adducts, probably mediated by impaired tubular reuptake since these adducts pass readily through the glomerular filter (molecular mass <500 Da). Renal clearances of AGE free adducts increase in type 1 diabetes with normoalbuminuria, compared to healthy controls, and may increase further in new onset microalbuminuria [20].

There was no correlation of any of the protein damage adduct residues of plasma protein nor concentrations of related free adduct with subsequent early GFR decline. This suggests that glycation, oxidation and nitration of plasma proteins may not be strongly linked mechanistically to early GFR decline. The same may apply for plasma concentrations of protein glycation, oxidation and nitration related free adducts – although the many influential factors affecting levels of these analytes - whole body flux of glycation, oxidation and nitration of proteins, rates of tissue proteolysis, glycation, oxidation and nitration adduct repair and free adduct renal clearance – may make a link difficult to discern.

The strengths of this study are: (a) analysis of an inception cohort of observed new onset microalbuminuria cases, (b) use of the best available methods for evaluation of markers of protein glycation, nitration and oxidation and analysis of a comprehensive range of protein damage adducts, and (c) discrimination between protein glycation, oxidation and nitration adducts in plasma protein and related free adducts – the former resistant to filtration and the latter readily filtered by the renal glomeruli [34]. However, the current study has potential limitations. Firstly, glycation, oxidation and nitration adduct residues of plasma protein may be formed and repaired beyond the systemic circulation – such as during the renal retrieval pathway for albumin [35]. This is, however, considered to have a minor influence except on repair of MetSO [36]. Similarly, glycation, oxidation and nitration free adducts are partly sourced from absorption from the gastrointestinal tract following digestion of ingested glycated, oxidized and nitrated proteins [37], [38]. This is usually a minor source for the free adducts studied herein as decrease in food consumption had a minor effect on glycation, oxidation and nitration free adduct concentration in diabetes [17]. Moreover, the finding of a subset of free adducts with lower plasma concentration implies that dietary exposure is an unlikely mechanism of early renal injury [39]. Secondly, the study has limited statistical power to resolve complex associations of multiple mechanisms of protein damage, development of microalbuminuria and early GFR decline. Thirdly, we have not studied similar effects in patients with type 2 diabetes which will be an important focus for future studies. It is interesting to note that a recent cross-sectional study of retinopathy in patients with type 1 diabetes of longstanding duration found a link between the presence of retinopathy and a combination of higher total plasma CEL and PENT adducts, but lower CML and FL [40].

We conclude that complex processes appear to influence levels of markers of plasma protein damage and plasma levels of related proteolysis product, free adducts, in patients with type 1 diabetes and microalbuminuria with and without early GFR decline. The effects observed likely relate to change in glomerular filter permeability of damaged proteins and change in tubular re-uptake of free adducts in microalbuminuria. Further research is required to evaluate involvement of these likely mechanisms of renal dysfunction.
